# Validation of the parents’ version of the KINDL^R^ and Kiddy Parents questionnaire in a South African context

**DOI:** 10.1186/s12955-024-02292-5

**Published:** 2024-09-11

**Authors:** Elmari Deacon, Esmé Jansen van Vuren, Elizabeth Bothma, Chanelle Volschenk, Ruan Kruger

**Affiliations:** 1 Optentia Research Unit, North-West University, Potchefstroom, South Africa; 2 Hypertension in Africa Research Team (HART), North-West University, Private Bag X6001, Potchefstroom, 2520 South Africa; 3 MRC Research Unit for Hypertension and Cardiovascular Disease, North-West University, Potchefstroom, South Africa

**Keywords:** Health related quality of life, KINDL^R^, Validation, South Africa, Children

## Abstract

**Background:**

This study aimed to assess the usefulness of the parent version of the KINDL^R^ and the additional items of the Kiddy Parents questionnaire in the South-African context and to validate it as an appropriate tool for measuring health-related quality of life (HRQoL).

**Method:**

The ExAMIN Youth SA study was designed to investigate lifestyle behaviours, including psychosocial factors that may adversely impact on cardiovascular health of children. Construct validity was examined by using exploratory and confirmatory factor analysis, while internal consistency was tested by Cronbach’s alpha. The final factor structure was confirmed by model fit indices.

**Results:**

The study included children (*n* = 1088) aged between 5 and 10 years in North-West, South Africa. The reliability coefficients of the original factors could not be reproduced in this data set, with the Cronbach’s alphas ranging between 0.46 and 0.78. With exploratory factor analysis, including the additional items, our data supported a 7-factor structure with acceptable internal consistency (Cronbach’s alpha: 0.68–0.79; Omega: 0.75–0.85) and acceptable model fit indices (CFI: 0.91; TLI: 0.90; RMSEA: 0.05; SRMR: 0.07). Two factors (emotional wellbeing and everyday functioning) further split into separate factors for positive and negative experiences related to each of these dimensions.

**Conclusion:**

We confirmed a new factor structure of the parent version of the KINDL^R^ and the additional items of the Kiddy Parents questionnaire, which can be used in the African context. Although the new factor structure has great overlap with the original structure, some items did not contribute to the factors as expected. Language and cultural differences between the original German group and the current South African study group resulted in a different factor structure.

**Supplementary Information:**

The online version contains supplementary material available at 10.1186/s12955-024-02292-5.

## Background

South African children are exposed to various adverse childhood experiences (ACEs) [19, 20] that can contribute to detrimental effects on health in later life [11, 22]. Major ACEs include three broad categories, i.e., (i) abuse (such as emotional, physical, and sexual), (ii) neglect (both emotional and physical), and (iii) household dysfunction (substance abuse, mental illness, domestic violence, criminal household member/s, and parental marital discord) [23]. Studies have shown strong associations between ACEs and cardiovascular disease (CVD) [17]. Experimental and human studies highlighted the role of childhood adversity in the development of hypertension in children [12, 14, 15, 23]. Since childhood hypertension tracks into adulthood and increases the risk of early CVD morbidity and mortality [1, 4, 29], the presence of ACEs in childhood adds to the burden of early adversity in the paediatric context.

Health-related quality of life (HRQoL) can act as a protective factor for children living in adversity [24] and refers to the overall well-being of a person in terms of health status, including physical, emotional, social and mental health [32, 33]. A better understanding of HRQoL can aid in identifying subgroups of children who are at risk for health problems, in determining the burden of a particular disease or disability, and in informing efforts aimed at prevention and intervention [13, 16]. In order to understand the context of HRQoL of children in South Africa, we need sound measures to assess HRQoL. The KINDL was developed by Bullinger et al. (1994), for use in clinical populations, but also with healthy children and adolescents [3]. It was originally intended to be a German-language measure as opposed to many other quality of life measures developed in English. The intention of the measure was to address the need for an appropriate measure to determine quality of life for youth.

The Exercise, Arterial Modulation and Nutrition in Youth South Africa (ExAMIN Youth SA) study [18] is an international collaborative study aligned with the Exercise and Arterial Modulation in Children study from Basel, Switzerland [8] in which similar methodologies were followed. This was done to enable comparative studies on lifestyle behaviours (such as physical activity and nutrition), but also psychosocial factors involved in early CVD development among South African children. At both baseline and follow-up, the parents of the children completed a set of health questionnaires, including the KINDL^R^, which was also administered in the Swiss EXAMIN YOUTH study. The KINDL^R^ assesses the HRQoL in children from age 3 years and older [3] and is completed by the parents of children and adolescents (aged 8–16 years). Although the KINDL^R^ is available in 31 languages, the norms are based on representative data from the German National Health Interview and Examination Survey for Children and Adolescents (KiGGS) [10]. While several tools exist to assess the level or degree of HRQoL in children and adolescents (including the KINDL^R^ survey), the usefulness or validity in the South African context is not yet known. Prior to comparing cross-continental cohorts, the KINDL^R^ therefore needs validation in a South African children’s cohort.

In this analysis, we evaluate the usefulness of the KINDL^R^ in South African children. The scale consists of 24 Likert-type items with six dimensions: physical well-being, emotional well-being, self-esteem, family, friends and everyday functioning (school in this case). A set of 22 additional items were also included as part of the version to provide additional information. In the German reference group, the original measure [26] showed relatively good internal consistency for self-report and parental version subscales with Cronbach’s alpha values around 0.70 (0.63 and 0.75 for self-report versions and 0.62 and 0.81 for parental version). In a study reporting on the psychometric properties of the KINDL^R^ [3], self-report internal consistency, measured by Cronbach’s alpha, varied between 0.54 and 0.73 for subscales. However, in all the incidences mentioned above, the Cronbach’s alpha for the total scale was good (0.84, 0.89 and 0.8 respectively). The lower reported internal consistency necessitates the investigation of the validation of the KINDL^R^, in every new context, especially in South Africa. Furthermore, as the KINDL^R^ have not been used in South Africa before, the validation thereof is needed to ensure good scientific outcomes using the tool. The validation of the KINDL^R^ in South Africa can add to the usefulness of this tool by knowing the psychometric properties and factor structure of the KINDL^R^ in developing countries. This will allow researchers to use the measure in different contexts and provide a valid measure to determine HRQoL in children from developing countries.

## Methods

### Study design, setting and participants

The ExAMIN Youth SA study is an analytical, multidisciplinary, observational cohort study which included 1,103 children (age 5–10 years) attending public primary schools in the North West province of South Africa [18]. The study sites were located within the Dr. Kenneth Kaunda district in two of the southern municipal areas namely JB Marks (Potchefstroom) and Matlosana (Klerksdorp). The majority of the population in these areas consist of Black (82%), with the remainder comprising 14% White, 4% Mixed-race, and 1% Indian individuals.

### Procedures

In the main study, data were collected on lifestyle behaviours (physical fitness/activity, dietary intake and psychosocial factors) that are likely to be involved in the early development of CVD among South African children. Cardiovascular related data included office blood pressure, pulse wave analysis, static retinal microvascular imaging, anthropometry as well as urine and saliva samples to measure biochemistry. Several questionnaires were administered among others the parent reports of the KINDL^R^ questionnaire to assess HRQoL.

### Measures

#### Demographics questionnaire

The general health and demographics questionnaire was used to obtain socio-demographic information that included personal (age, sex and ethnicity) and family (home language) information.

#### KINDLR questionnaire

Parents were asked to complete the KINDL^R^ questionnaire [26] on a 5-point Likert scale that ranges from 1 (never) to 5 (all the time). The original sub-scales of the questionnaire that were used in the study consists of 24 items that are associated with six sub-scales as shown in Supplementary Table 1. These sub-scales were physical well-being (e.g., “my child felt ill, my child felt strong and full of energy”), emotional well-being (e.g., “my child had fun and laughed a lot, my child felt alone’’), family (e.g., “my child got on well with us as parents,” “we quarrelled at home”), friends (e.g., “my child got along well with his friends,” “my child felt different from other children’’), and everyday functioning at school (e.g., “my child easily coped with schoolwork,” “my child worried about his/her future”). Questions that were negative in nature were recoded before combining the subscales in order to compute a mean total score with higher scores reflecting a higher QoL. For the purposes of validating the measure in this study, different subscales were determined that also included the additional items of the Kiddy-KINDL^R^ questionnaire (Supplementary Table 2), as described in the statistical analyses section below.

### Statistical analysis

The data were formatted for use in the statistical software programmes IBM^®^ SPSS^®^ 27 (IBM Corporation; Armonk, New York, USA) and Mplus 8.6 [21]. Frequency analysis was done, and descriptive statistics (including means and standard deviations) were calculated using IBM^®^ SPSS^®^ 27. After inspecting the normality of the data distribution, further analyses were completed using latent variable modelling in Mplus 8.6.

### Factor analysis

A confirmatory factor analysis (CFA) serves to assess the proposed factor structure’s fit to the data of a specific sample. As the KINDL^R^ is an existing measure that has been validated in several contexts, the original factor structure was used as a starting point to evaluate the fit of the data to the model. For the six subscales, each contained four questions and were specified as follows: Physical well-being, Emotional well-being, Self-esteem, Family, Friends, and Everyday functioning at school, with 22 additional items pertaining to other characteristics of illness [26]. A first CFA was conducted based on the six defined subscales as mentioned, a second CFA with seven factors including the additional items as a single variable, namely Illness, a third CFA with a one-factor structure including only the first 24 items, and a final CFA with a one-factor structure including all 46 items. None of the CFAs achieved acceptable fit without issues, and, therefore, it was decided to conduct an exploratory factor analysis (EFA) to identify other possible underlying factor structures within the data. The dataset was randomly divided into two equal sets of data: one to use for the EFA, and the other for a CFA to confirm the suggested possible factor structure(s) identified through the EFA as a validation set. Due to splitting the dataset at random, sample dependence was avoided. In order to evaluate model fit, the following indices were utilised: the Chi-square (χ^2^) value and its degrees of freedom (*df*) – the lower the value, the better the fit; the Root Mean Square Error of Approximation (RMSEA) – should be < 0.08; the Comparative Fit Index (CFI) and Tucker-Lewis Index (TLI) – acceptable fit > 0.90; excellent fit > 0.95; and the Standardised Root Mean Square Residual (SRMR) – should be < 0.08. When similar models are compared, the Akaike Information Criterion (AIC) and sample-size Adjusted Bayes Information Criterion (ABIC) are used, with the lowest AIC and ABIC values indicating better fit [21, 34].

### Reliability measures

The internal consistency, or reliability, of the data was calculated in two ways: Cronbach’s alpha (α) [5] that assumes equal contribution by all included items, and Omega (Ω) [9], a composite reliability coefficient allowing for a difference in item weights. Although the calculation of the two measures of internal consistency differs, they are both acceptable at values above 0.70 [5, 9].

### Ethical considerations

Data collection procedures were conducted in a school-based setting with endorsement of the district’s Department of Education. The study obtained ethical approval (NWU-00091-16-A1) from the Health Research Ethics Committee of the North-West University and was registered on 12 August 2019 at ClinicalTrials.gov (NCT04056377). Written informed consent (for children above 7 years of age), assent (for children under 7 years of age) and parental/guardian permission were obtained prior to data collection with consent for publication and further use of data.

## Results

### Sample

**Fig. 1:**
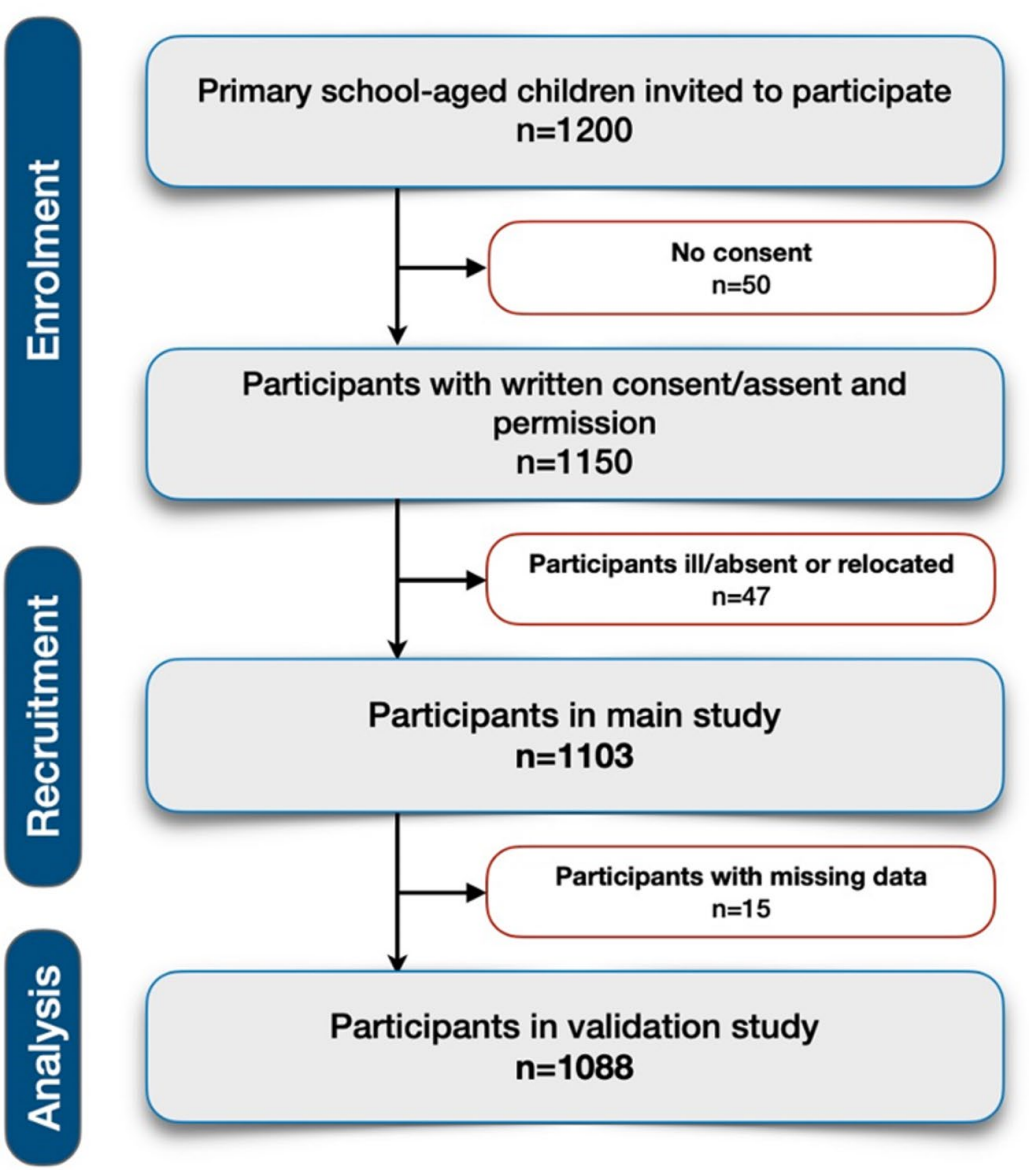
The ExAMIN Youth SA study design and number of included and excluded participants

Public primary school-aged (above 5 and under 10 years) boys and girls (*n* = 1,200) were invited to participate in the study. Participation was voluntarily and almost 96% (*n* = 1,150) provided written assent or consent along permission from the primary caregivers of the child. At the time of screening, 47 children were ill/absent or had relocated. All the participants in the main study (*n* = 1,103) were eligible for, and included in, this validation study. However, 15 children had missing data on all the variables reported in this analysis. Therefore, a total of *n* = 1,088 was included in this validation study (Fig. 1).

The characteristics of the participants included in this study are shown in Table 1. There were more females (*n* = 590) than males (*n* = 496) included in the final sample. Most of the children were either 7 (*n* = 364) or 8 (*n* = 322) years old. The children mainly spoke either Afrikaans (*n* = 444) or Setswana (*n* = 390), with isiZulu and languages not specified spoken the least, with only 8 children indicating each. The children were spread across four grades: Grade R – 161; Grade 1–360; Grade 2–366; and Grade 3–102.

**Table 1:** Characteristics of the participants (*n* = 1088)

Item	Category	Frequency	Percentage
Ethnicity	Black	597	54.9
	White	468	43.0
	Other	23	2.1
Sex	Female	590	54.3
	Male	496	45.7
Age - Years	5 years	85	8.0
	6 years	273	25.6
	7 years	364	34.1
	8 years	322	30.2
	9 years	18	1.7
	10 years	4	0.4
Home language	Setswana	390	39.2
	Sesotho	66	6.6
	isiZulu	8	0.8
	isiXhosa	61	6.1
	Afrikaans	444	44.7
	English	17	1.7
	Other	8	0.8
Grade	R	161	16.3
	1	360	36.4
	2	366	37.0
	3	102	10.3

### Confirmatory and exploratory factor analysis

The KINDL^R^ was used as the starting point for evaluating fit to the data. The results are reported in Table 2. Most of the fit indices did not meet the criteria for acceptable fit; the CFI and TLI values, were low. The reported reliability coefficients of the original factors could not be reproduced in this data set. The Cronbach’s alphas were found to be as follows: Physical well-being: α = 0.60; Emotional well-being: α = 0.54; Self-esteem: α = 0.78; Family: α = 0.49; Friends: α = 0.59; and Everyday Functioning (school): α = 0.46.

Since none of the four models achieved acceptable fit, we conducted an EFA on all items, and in order to obtain as much information as possible, we included the additional items, thus a total number of 46 items. Any variable should preferably contain at least three items, meaning that there might possibly have been up to 15 factors present in the data. To determine the optimal number of possible factors, Eigen-values were used, with values above 1.00 indicating that a certain number of items have enough in common to possibly represent one factor. These values showed that only 11 factors would be viable and an EFA was specified in Mplus 8.6 to extract possible solutions from one to eleven factors.

The resulting possible factor structures were evaluated for theoretical validation and a final possible factor structure was suggested/approved by a team of experts. Only items 29 and 38 were excluded, due to their extremely low loadings on all possible factors. This factor structure was used in a CFA to confirm whether it would fit the data. The final proposed factor structure consisted of seven factors: Positive everyday functioning (items 12, 21, 22, 33, 38, 43); Negative affect (emotional wellbeing; items 6, 7, 8, 15, 16, 20, 25, 28, 31, 34, 36, 39, 44, 45, 46); Physical well-being (items 1, 2, 3, 41); Positive affect (emotional wellbeing; items 4, 5, 13, 14, 26, 29, 30, 32, 37, 40, 42); Self-esteem (items 9, 10, 11); Friends (items 17, 18, 19, 27, 35); and Negative everyday functioning (items 23, 24), summarised in Table 3.

**Table 2:** Fit statistics of CFAs with original factor structures

Model	AIC	ABIC	c^2^	df	RMSEA	CFI	TLI	SRMR
Six-factor (original)	55819.69	55965.44	1176.74	237	0.07	0.78	0.75	0.08
One-factor (24 items)	56910.12	57259.40	2033.84	252	0.09	0.59	0.55	0.09
Seven-factor (including Illness)	107417.42	107684.12	4256.57	968	0.06	0.64	0.61	0.09
One-factor (46 items)	108673.06	108904.54	5254.19	989	0.07	0.53	0.51	0.09

AIC = Akaike Information Criterion; ABIC = Sample-size Adjusted BIC; χ² = chi-square; df = degrees of freedom; MLR-adjusted χ² = Maximum Likelihood Robust adjusted χ²; RMSEA = Root Mean Square Error of Approximation; CFI = Comparative Fit Index; TLI = Tucker-Lewis Index; SRMR = Standardized Root Mean Square Residual

**Table 3:** Summary of the newly developed sub-scales of the KINDL^R^ in a South African context

New sub-scales	Item number	Item description	Item’s original sub-scale
Positive everyday functioning	12 21 22 33 43	My child had lots of good ideas My child easily coped with schoolwork My child enjoyed the school lessons My child was alert and able to concentrate well My child succeeded at everything he set out to do	Self-esteem Everyday functioning at school Everyday functioning at school Additional item of the Kiddy-KINDL^R^ Additional item of the Kiddy-KINDL^R^
Negative affect emotional well-being	6 7 8 15 16 20 25 28 31 34 36 39 44 45 46	My child didn’t feel much like doing anything My child felt alone My child felt scared or unsure of itself We quarrelled at home My child felt that I was bossing him around My child felt different from other children My child was moody and whined a lot My child felt under pressure My child kept bursting into tears My child was easily distracted and absent- minded Had to give my child a telling-off My child was nervous and fidgety My child became dissatisfied easily My child cried bitterly My child lost his temper quickly	Emotional well-being Emotional well-being Emotional well-being Family Family Friends Additional item of the Kiddy-KINDL^R^ Additional item of the Kiddy-KINDL^R^ Additional item of the Kiddy-KINDL^R^ Additional item of the Kiddy-KINDL^R^ Additional item of the Kiddy-KINDL^R^ Additional item of the Kiddy-KINDL^R^ Additional item of the Kiddy-KINDL^R^ Additional item of the Kiddy-KINDL^R^ Additional item of the Kiddy-KINDL^R^
Physical well-being	1 2 3 41	My child felt ill My child had a headache or tummy-ache My child was tired and worn-out My child complained of being in pain	Physical well-being Physical well-being Physical well-being Additional item of the Kiddy-KINDL^R^
Positive affect emotional well-being	4 5 13 14 26 30 32 37 40 42	My child felt strong and full of energy My child had fun and laughed a lot My child got on well with us as parents My child felt fine at home My child had a healthy appetite My child romped around and was very active My child was cheerful and in a good mood I praised my child My child was lively and energetic My child was sociable and out- going	Physical well-being Physical well-being Family Family Additional item of the Kiddy-KINDL^R^ Additional item of the Kiddy-KINDL^R^ Additional item of the Kiddy-KINDL^R^ Additional item of the Kiddy-KINDL^R^ Additional item of the Kiddy-KINDL^R^ Additional item of the Kiddy-KINDL^R^
Self-esteem	9 10 11	My child was proud of himself My child felt on top of the world My child felt pleased with himself	Self-esteem Self-esteem Self-esteem
Friends	17 18 19 27 35	My child did things together with friends My child was liked by other kids My child got along well with his friends I managed to show patience and understanding towards my child My child enjoyed being with other children	Friends Friends Friends Additional item of the Kiddy-KINDL^R^ Additional item of the Kiddy-KINDL^R^
Negative everyday functioning	23 24	My child worried about his future My child was afraid of bad marks or grades	Everyday functioning at school Everyday functioning at school
		**Items that did not load on any sub-scale**	
	29 38	My child slept soundly My child had problems with teachers, kindergarten staff or other child-minders	Additional item of the Kiddy-KINDL^R^ Additional item of the Kiddy-KINDL^R^

### Model fit indices

The proposed factor structure was specified in Mplus (Muthén & Muhén, 1998–2022) for estimation in a CFA. As can be seen in Table 4, only the TLI incremental fit index originally did not reach its cut-off value of 0.90. Factor loadings and modification indices were inspected for possible issues. No problems were found with any factor loadings, as they were all above the cut-off value of 0.35. Two modification indices indicated that two sets of items had a lot in common: Items 18 and 19, and items 31 and 45. Their error variances were allowed to correlate, and the final model achieved acceptable fit, without any further items having to be left out.

### Correlations and internal consistency

The final 7-factor structure was used to determine descriptive statistics, reliability, and correlations. These are reported in Table 5. The means of all the factors were quite close to each other, except Factor 6: Friends that had a higher mean, and Factors 1 to 5 had acceptable standard deviations (between − 1.00 and + 1.00), meaning that the participants’ scores on these factors were closely distributed around the mean. Only Factor 7 had a lower mean and a large standard deviation (M = 3.53, SD = 1.19), thus indicating less similarity in their points of view. Both the Cronbach’s alpha and the Omega coefficients of reliability were calculated, as alpha is based on the weights of items being equal, while Omega accounts for different weights items might add to the measurement of a specific factor. Overall, the reliability coefficients were extremely close in their measurement of internal consistency, and good reliability (> 0.70) were indicated for most factors. The only factor that did not display the preferred level of reliability was Factor 7: Negative everyday functioning, with α = 0.68.

Between Factors 1 to 5, all correlations were found to be either significant (*p* < 0.05) or highly significant (*p* < 0.01). The relationships with the largest effects were between Factor 4: Positive affect and Factor 5: Self-esteem (*r* = 0.83), Factor 4: Positive affect and Factor 6: Friends (*r* = 0.82), and Factor 1: Positive everyday functioning and Factor 4: Positive affect (*r* = 0.79). Factor 7: Negative everyday functioning was indicated as having the least significant number of relationships with the other factors, specifically with factors 1: Positive everyday functioning (*r*=-0.02), 4: Positive affect (*r*=-0.06), and 5: Self-esteem (*r*=-0.02). Other significant correlations were shown to have only a small effect, with the smallest being between Factor 6: Friends and Factor 7: Everyday negative functioning (*r* = 0.17), and Factor 1: Positive everyday functioning and Factor 3: Physical well-being (*r* = 0.17).

**Table 4:** Fit statistics of CFA: proposed factor structure and final factor structure

Model	AIC	ABIC	χ^2^	df	RMSEA	CFI	TLI	SRMR
EFA 7-factor model (44 items)	45484.56	45725.53	2045.00	881	0.05	0.90	0.89	0.07
Final 7-factor model (44 items)	45484.56	45725.53	2002.01	879	0.05	0.91	0.90	0.07

AIC = Akaike Information Criterion; ABIC = Sample-size Adjusted BIC; χ^2^ = chi-square; df = degrees of freedom; MLR-adjusted χ^2^ = Maximum Likelihood Robust adjusted χ^2^; RMSEA = Root Mean Square Error of Approximation; CFI = Comparative Fit Index; TLI = Tucker-Lewis Index; SRMR = Standardized Root Mean Square Residual

**Table 5:** Descriptive statistics, reliability coefficients, and correlations

Variable		M	SD	α	Ω	1	2	3	4	5	6
1.	Positive everyday functioning	4.03	0.67	0.79	0.79	-					
2.	Negative affect	3.92	0.56	0.85	0.85	-0.42†**	-				
3.	Physical well-being	3.98	0.72	0.73	0.74	0.17**	-0.60‡**	-			
4.	Positive affect	4.15	0.50	0.75	0.75	0.79‡**	-0.42†**	0.25**	-		
5.	Self-esteem	4.01	0.73	0.77	0.78	0.69‡**	-0.40†**	0.23**	0.83‡**	-	
6.	Friends	4.33	0.57	0.78	0.78	0.66‡**	-0.45†**	0.21**	0.82‡**	0.69‡**	-
7.	Negative everyday functioning	3.53	1.19	0.68	-	-0.02	-0.39†**	0.21**	-0.06	-0.02	0.17**

Symbol denotes significance for: *p < 0.05; **p < 0.01; †*r* > 0.30; ‡*r* > 0.50

## Discussion

This study investigated the factor structure of the KINDL^R^ in the South African context. The findings confirm the basic factors structure of the original measure but refines the items and indicates that factors divided further in this context. The final factors were well supported by model fit indices and showed good reliability, and strong correlations between factors.

We used the KINDL^R^ (8 to 16-year-olds) Parents’ Version and the additional items of the Kiddy-KINDL^R^ (4 to 7-year-olds) Parents’ Version. The first items for the Kiddy-KINDL^R^ Parents’ Version are mostly similar with some smaller differences, hence the additional questions of the Kiddy-Parents were added. The first step in exploring the factors structure in this context was to make sure that all the items contribute to the measure. During this step items 29 and 38 were excluded due to extreme low loadings. When looking more closely at the items, it is clear that the terms used could be confusing in the current context and would be more applicable to the cultural context it was developed in. The specific items (item 29 – My child slept soundly; 38 – My child has problems with teachers, kindergarten staff or other child-minders) could easily be misunderstood due to different expressions in language.

The new factor structure has strong similarities with the original structure, as is evident in Table 3. What is interesting to note is that the additional items (25–46) fit very well within the original structure and contributed towards the strength of the factors. The main differences in factors are as follows: Physical well-being was presented by items 1–4, while item 4 moved to the cluster of emotional well-being (positive affect) in the new factor structure. Emotional well-being (items 5–8) divided into two factors, namely positive affect (items 4, 5, 13, 14 from the original scale and 26, 30, 32, 37, 40, and 42 from the additional items), and negative affect (items 6, 7, 8, 15, 16, and 20 from the original scale and items 25, 28, 31, 34, 36, 39, 44, 45, 46 from the additional items). It is interesting to note that a separate family dimension factor was not present in the new factor structure, with all the items of this scale being taken up into the emotional well-being scales. This coincides with the findings of various studies that shows the positive or detrimental effects of different family processes on the emotional reactions and adaptions of children [2, 30].

The self-esteem scale remained mostly similar, except for item 12, which moved to the positive everyday functioning scale. The subscale of everyday functioning showed the biggest differences from the original scale. In the original measure, the scale focus on everyday functioning at school, while the new factors had a broader focus and was strengthened by a number of additional items. Positive everyday functioning includes two items form the original scale (items 21 and 22), with 2 from the additional items (33 and 43). Negative everyday functioning now included only 2 items, namely 23 and 24, but the items showed such strong loadings that it could not be removed from the list of possible factors. It’s psychometric properties were problematic, however, and the factor might need some alteration for future use. In the last factor, Friends, three of the original items remained (item 17, 18 and 19), with one item moving to negative affect emotional well-being (item 20). Two items were added from the additional items (item 27 and 35).

It is important to note the improvement in reliability indicators when using the 7-factor structure as proposed in the study, with Cronbach’s alpha’s varying between 0.68 and 0.79 and Omega coefficients between 0.75 and 0.85. The new structure seems to be more reliable, especially in the South African context as previous studies reported Cronbach’s alphas of between 0.54 and 0.81. In this study, EFA and CFE confirmed an adjusted factor structure with adjusted items to measure the same theoretical constructs as proposed by the original measure. The adaptation was hence concept driven, with changes being made to content because of differences in culture-specific concepts. In future, further investigation could explore linguistic equivalence between different cultures [6].

Correlations between the factors of the final structure further confirmed the coherence of the relationships between the different factors. Positive correlations between the factors, positive everyday functioning, positive affect, physical wellbeing, self-esteem and friends are supported by the literature [7, 25, 28]. Evidence also exists [27] that supports the negative correlations observed between negative affect, positive everyday functioning, positive affect, self-esteem, and friends. The interpretation of the correlations between factors supports the integrated nature of well-being in children. In this study, it was evident that happier children (positive affect), had higher self-esteem (positive correlation with self-esteem), experienced better adjustment to everyday challenges (positive correlation to positive everyday functioning) and had more friends (positive correlation with friends). Furthermore, children who struggled to adjust (negative everyday functioning) experienced fewer positive emotions (negative correlation with positive affect) and was also not as sure of themselves (negative correlation with self-esteem). It is interesting to note that negative everyday functioning had a negative correlation with negative affect, which could indicate that learners who struggle might not necessarily experience negative affect. These learners should be identified and assisted to prevent the possible development of negative emotions and feelings of sadness and worthlessness.

### Strengths and limitations

This study contributes to HRQoL in that it is the fits attempt to validate the KNDL^R^ in a developing context, using a large sample. We do acknowledge that a larger pilot study or panel of experts checking the appropriateness of the language of the items would have been beneficial before the study commenced. In future studies, this will be an important consideration to be more proactive in identifying and addressing problematic items. Although the new factor structure shows an improvement on the reliability of the measure and the factor structure is sound, Factor 7 (negative everyday functioning) should be further investigated as this factor might be problematic with only 2 items contributing to it. By validating the use of this measurement tool in the South African context, we are now able to use the proposed subscales to test across different cultures and regions in South Africa. Our proposed subscales seem to be well fitted in this context, while further work is still to be done to determine its generalizability. In this study we did not do differential item functioning which uses statistical procedures to compare test results of test-takers with the same ability but who belongs to different cultural or language groups. By investigating this in future studies, the investigation could further assist in understanding the outcomes of the measure.

### Implications and conclusions

The importance of the validation of measure in the context it will be used, was again emphasized in this study. Although the original factor structure is strongly resembled in the proposed structure, some smaller changes was made that improved the overall reliability of the measure. Within the South African context, the influence of language and cultural context should always be taken into consideration. As South-Africa is a country grappling with unequal distribution of resources and a failing health system [31], the assessment of HRQoL is important for policy making. As such, the parent version of the KINDL^R^ and the additional items of the Kiddy Parents questionnaire, could be a helpful tool in the evaluation of the health care needs of a community, aiming to develop strategic health care plans to address CVD in youth.

In conclusion, the new factor structure identified in this validation study indicated stronger reliability indices in the South African setting and is encouraged to be used in similar population settings.

## Electronic supplementary material

Below is the link to the electronic supplementary material.

Supplementary Material 1

Supplementary Material 2

## Data Availability

No datasets were generated or analysed during the current study.

## Abbreviations

ABIC: Adjusted Bayes Information Criterion.
ACE: Adverse Childhood Experiences
AIC: Akaike Information Criterion
CVD: Cardiovascular Disease
CFI: Comparative Fit Index
CFA: Confirmatory Factor Analysis
ExAMIN Youth SA: Exercise, Arterial Modulation and Nutrition in Youth South Africa
EFA: Exploratory Factor Analysis
KiGGS: German National Health Interview and Examination Survey for Children and Adolescents
HRQoL: Health-Related Quality of Life
NRF: National Research Foundation
RMSEA: Root Mean Square Error of Approximation
SA MRC: South African Medical Research Council
SRMR: Standardised Root Mean Square Residual
TLI: Tucker-Lewis Index
