# Supplementary material for: Validation of the parents’ version of the KINDLR and Kiddy Parents questionnaire in a South African context
Source: Health Qual Life Outcomes. 2024 Sep 11;22:77. doi: 10.1186/s12955-024-02292-5 (PMC11389106; doi:10.1186/s12955-024-02292-5)
Supplement: Supplementary file 1 — Supplementary Material 1 [file 12955_2024_2292_MOESM1_ESM.docx]

**Supplementary Table 1.** Items that are associated with the original sub-scales of the original KINDL^R^.

| **Original KINDL^R^ sub-scales** | **Items forming the sub-scale** |
| --- | --- |
| **Physical well-being** | 1. my child felt ill 2. my child had a headache or tummy-ache 3. my child was tired and worn-out 4. my child felt strong and full of energy |
| **Emotional well-being** | 1. my child had fun and laughed a lot 2. my child didn't feel much like doing anything 3. my child felt alone 4. my child felt scared or unsure of her-/ himself |
| **Self-esteem** | 1. my child was proud of him-/herself 2. my child felt on top of the world 3. my child felt pleased with him-/ herself 4. my child had lots of good ideas |
| **Family** | 1. my child got on well with us as parents 2. my child felt fine at home 3. we quarrelled at home 4. my child felt that I was bossing him/her around |
| **Friends** | 1. my child did things together with friends 2. my child was liked by other kids 3. my child got along well with his/her friends 4. my child felt different from other children |
| **Everyday functioning at school** | 1. my child easily coped with schoolwork 2. my child enjoyed the school lessons 3. my child worried about his future 4. my child was afraid of bad marks or grades |
