# Supplementary material for: Validation of the parents’ version of the KINDLR and Kiddy Parents questionnaire in a South African context
Source: Health Qual Life Outcomes. 2024 Sep 11;22:77. doi: 10.1186/s12955-024-02292-5 (PMC11389106; doi:10.1186/s12955-024-02292-5)
Supplement: Supplementary file 2 — Supplementary Material 2 [file 12955_2024_2292_MOESM2_ESM.docx]

**Supplementary Table 2.** Additional items of the Kiddy version of the KINDL^R^ questionnaire.

| **Additional items of the Kiddy-KINDL^R^** |
| --- |
| 25. my child was moody and whined a lot  26. my child had a healthy appetite  27. I managed to show patience and understanding towards my child  28. my child felt under pressure  29. my child slept soundly  30. my child romped around and was very active  31. my child kept bursting into tears  32. my child was cheerful and in a good mood  33. my child was alert and able to concentrate well  34. my child was easily distracted and absent- minded  35. my child enjoyed being with other children  36. I had to give my child a telling-off  37. I praised my child  38. my child had problems with teachers, kindergarten staff or other child-minders  39. my child was nervous and fidgety  40. my child was lively and energetic  41. my child complained of being in pain  42. my child was sociable and out- going  43. my child succeeded at everything he set out to do  44. my child became dissatisfied easily  45. my child cried bitterly  46. my child lost his temper quickly |
